# Comparison of intra-articular injections of Hyaluronic Acid and Corticosteroid in the treatment of Osteoarthritis of the hip in comparison with intra-articular injections of Bupivacaine. Design of a prospective, randomized, controlled study with blinding of the patients and outcome assessors

**DOI:** 10.1186/1471-2474-11-264

**Published:** 2010-11-16

**Authors:** Sascha Colen, Michel PJ van den Bekerom, Johan Bellemans, Michiel Mulier

**Affiliations:** 1 University of Leuven, Department of Orthopaedic Surgery, Weligerveld 1, 3212 Pellenberg, Belgium; 2 Academic Medical Centre Amsterdam, Department of Orthopaedic Surgery, Meibergdreef 15, 1105 Amsterdam Zuid-Oost, Amsterdam, The Netherlands

## Abstract

**Background:**

Although intra-articular hyaluronic acid is well established as a treatment for osteoarthritis of the knee, its use in hip osteoarthritis is not based on large randomized controlled trials. There is a need for more rigorously designed studies on hip osteoarthritis treatment as this subject is still very much under debate.

**Methods/Design:**

Randomized, controlled trial with a three-armed, parallel-group design. Approximately 315 patients complying with the inclusion and exclusion criteria will be randomized into one of the following treatment groups: infiltration of the hip joint with hyaluronic acid, with a corticosteroid or with 0.125% bupivacaine.

The following outcome measure instruments will be assessed at baseline, i.e. before the intra-articular injection of one of the study products, and then again at six weeks, 3 and 6 months after the initial injection: Pain (100 mm VAS), Harris Hip Score and HOOS, patient assessment of their clinical status (worse, stable or better then at the time of enrollment) and intake of pain rescue medication (number per week). In addition patients will be asked if they have complications/adverse events. The six-month follow-up period for all patients will begin on the date the first injection is administered.

**Discussion:**

This randomized, controlled, three-arm study will hopefully provide robust information on two of the intra-articular treatments used in hip osteoarthritis, in comparison to bupivacaine.

**Trial registration:**

NCT01079455

## Background

Socioeconomic costs have, in general, escalated dramatically in the last ten years [1] with osteoarthritis (OA) being one of the most important causes for this increase. OA is a very common problem in the older population [2,3] and affects almost 5% of people over 65 years of age [4]. OA of the hip is one of the most common causes of functional impairment in the elderly [5,6]. Despite the immense impact of this disease on many people very few effective, non-surgical options are available to handle it. In this regard, (intra-articular) pharmacological treatment is of special interest for two reasons: on the one hand, as basic agent for the relief of pain and pain flares in more acute situations, and on the other, as a way to postpone any surgical intervention by improving the patients' subjective quality of life. In contrast to the knee, the access to the intra-articular compartment of the hip is rather difficult as a result of which injection therapy for hip OA has not been commonly used in the past.

Nowadays, two generally accepted products are used for intra-articular injection in the hip: corticosteroids (CS) and hyaluronic acid (HA). The effects of CS in hip OA have been demonstrated in several controlled studies [7,8]. However its widespread use remains controversial because of its short-term effect and reports of adverse effects following injection [8,9].

The effect of intra-articular HA in the treatment of hip OA has not been fully elucidated. Although studies have shown that HA injections provide prolonged relief from symptoms in patients with knee OA [10-12], some controversy exists over the benefit of HA in the treatment of hip OA. The conclusion of a literature review highlighted the absence of placebo-controlled studies in the treatment of hip OA with intra-articular HA or its derivatives [13]. This means that the role of intra-articular HA in the symptomatic treatment of hip OA cannot be determined conclusively. Nevertheless published data suggest that intra-articular injections of HA in hip OA may be effective. Another literature review showed a relatively low level of evidence of the included studies, although the authors concluded that intra-articular injection of HA, performed under fluoroscopic or ultrasound guidance seems to be an effective treatment and may be an alternative treatment of hip OA [14]. Double-blind, controlled studies with a higher level of evidence are required to confirm these data.

The effectiveness of HA in comparison to CS is also not very clear. One study compared the results of HA with that of CS in patients with hip OA, but a higher number of patients was needed to clarify their conclusion [15]. Most published studies do not have a reliable control group, have too short follow-up periods, or a bias in outcome measure [16,17].

The aim of our randomized, controlled study is to compare the effects of intra-articular injections of HA and CS with a control group, which will receive an intra-articular infiltration with bupivacaine, in patients with painful hip OA. To our knowledge there are no clinical studies which compare the effects of HA, CS and bupivacaine in patients with hip OA.

## Hypothesis

(1) There are differences in functional outcome [differences of more than 10 points using the Harris Hip Score (HHS): [18]] and the Visual Analog Score [VAS: difference of more than 10 mm [19]] between the treatment groups (HA and CS infiltration) and the control group (Bupivacaine) in favour of the treatment groups, at 6 months follow-up in the treatment of symptomatic hip OA patients.

(2) There are differences in secondary outcome measures [Hip disability and Osteoarthritis Outcome Score (HOOS) [20]] between the treatment groups and the control group in favour of the treatment groups, at 6 weeks, 3 and 6 months follow-up in the treatment of symptomatic hip OA patients.

(3) There are differences in functional outcome and the VAS between the treatment groups and the control group in favour of the treatment groups, at 6 weeks and 3 months follow-up in the treatment of symptomatic hip OA patients.

## Methods/design

All patients will be evaluated in the outpatient clinic of our department. After checking the inclusion and exclusion criteria (table 1), eligible patients will be informed about the study and the study design. They will also be well informed about the advantages and disadvantages of participation and the adverse effects of the products. They will be given time to decide if they wish to participate and will be told that their treatment at the centre would not be jeopardized if they declined to participate.

**Table 1:** Eligibility criteria

Inclusion criteria:
1. male and female patients aged between 30 and 70 years
2. pain (VAS) of at least 30 mm on the day of inclusion.
3. radiographic evidence of OA of the hip (Kellgren-Lawrence grading scale 1-3),
4. chronic pain for at least 3 months prior to study entry (day 0),
5. dissatisfaction with previous attempts at non-operative management including nonsteroidal anti-inflammatory drugs.

**Exclusion criteria:**

1. Kellgren and Lawrence grade 4,
2. an intra-articular hip injection (with any corticosteroid, hyaluronic acid preparation or other) within the previous three months,
3. rapid destructive hip OA.
4. a history of crystalline arthropathy or inflammatory arthritis, neuropathic arthropathy,
5. current other problem in the affected extremity,
6. allergy or hypersensitivity to any of the study medications or to contrast solutions.

Eligible patients that accept to participate will be asked to sign the informed consent form and will be assigned an inclusion number. Approximately 1 month later they will have their treatment (= infiltration).

At day 0 [the day of the infiltration (table 2)] demographic data including age, height, weight, body-mass index (BMI), target hip joint, and the use of nonsteroidal anti-inflammatory drugs will be recorded. The type of treatment the patient will receive will be checked. A nurse working at our day hospital will check the inclusion number and from the internet site http://www.randomizer.org she will, on the day of infiltration, obtain a randomized number ranging from 1 to 3 which corresponds to the treatment injection to be given.

**Table 2:** Flowchart

Day ??	Day of inclusion:	Demographic data, clinical history, concomitant medication, informed consent, inclusion/exclusion criteria.
Day 0	Day of infiltration:	Pain (VAS), HOOS and HHS-score, randomization and infiltration.
Day 1-14	Diary:	Daily VAS and use of pain killers per day.
Week 6	Second consultation:	VAS, HOOS and HHS-score.
Week 12	Third consultation:	VAS, HOOS and HHS-score.
Week 26	Fourth consultation:	VAS, HOOS and HHS-score, end follow-up.

Approximately 315 patients will be included in the study. (see: Planned statistical analysis and sample size calculation).

Injection of the study drugs will be performed under sterile conditions by the same experienced orthopaedic surgeon and senior author (MM) in all patients. After skin cleaning a lumbar puncture needle is inserted in a lateral approach. Layer by layer local anaesthesia is performed using lidocaine 1%. Arthrocentesis is carefully performed prior to each injection to remove any effusion. Iodinated contrast agent Ultravist^® ^(Schering, Berlin, Germany) will be injected. The needle positioning into the joint cavity will be fluoroscopically controlled when the contrast is injected. When the needle is in the correct position the injection will be performed. Another option to perform the injection in the hip is using ultrasound [21,22]. The skin is cleaned and a lumbar puncture needle is inserted in an anterior approach. Layer by layer local anaesthesia is performed using lidocaine 1%. Arthrocentesis is performed followed by the injection of the assigned study product. After resting for 2 hours, the patient is allowed to walk and to return home. The patient is advised to rest at home until the next morning. Oral symptomatic slow acting drugs for OA (for example glucosamine and chondroitin) are authorized if they are taken at a stable dose for more than 3 months prior to inclusion in the study. These drugs are continued at a stable dose during the study treatment period.

There will be 3 different groups of treatment:

1. Infiltration of the hip joint with Hyaluronic Acid (Ostenil plus 40 mg/2ml).

2. Infiltration of the hip joint with Corticosteroids (Depo-Medrol 80 mg/2ml).

3. Infiltration of the hip joint with 0.125% Bupivacaine (0.125% Marcaine 2 ml).

All the products used in this study are already used in our department. To date, we have never had problems using these products.

"Post-injection" data collection will be performed by a person who is blinded to the treatment received by each patient and who is skilled in the administration of the study outcome instruments.

There will also be a diary to be filled in by each included patient for the first two weeks after the infiltration to assess if the treatments have an effect on hip OA. The diary will include a VAS scale (0-100 mm) for each day. Patients will be required to fill in their VAS score and also the number of pain killers used each day. Side effects will also be reported.

In summary we can conclude that both the patient and the person who will collect the "post-injection" data will not know which kind of infiltration the patient has received.

The findings on the initial radiographs will be graded by a musculoskeletal radiologist, experienced in reviewing orthopedic X-rays using the Kellgren and Lawrence grades.

### Further information on the study products

#### Hyaluronic acid

One of the non-operative options used for reducing pain and maintaining hip mobility in patients with hip OA is viscosupplementation. Viscosupplementation is the administration of HA preparations into the affected joint to supplement the viscoelasticity of the diseased synovial fluid.

The underlying mechanisms of action of HA in osteoarthritic joints are not completely understood. There is increasing evidence to indicate that clinical efficacy is mediated through several pathways: anti-inflammatory effects, anti-nociceptive effects, normalization of endogenous HA synthesis and chondroprotection [23].

It takes less than a day to clear the injected HA from the osteoarthritic joint [24]. To increase the average intra-articular half-life, TRB Chemedica produced Ostenil Plus which will be used in our study. Ostenil Plus has a higher a concentration of hyaluronic acid (40 mg/2 ml) which is twice as much as previously used in the product Ostenil. In addition Ostenil Plus contains 10 mg Mannitol, an antioxidant which protects hyaluronic acid molecules against free radicals and inhibits the degradation of HA [25]. Ostenil Plus is a low molecular weight hyaluronic acid (LMW HA) (1.6 million Dalton).

One meta-analysis showed no important evidence for a clinically relevant benefit of a high molecular weight hyaluronic acid (HMW HA) compared with LMW HA in patients with OA of the knee [26]. The authors concluded that given the lack of a superior effectiveness of HMW HA over LMW HA and the increased risk of local adverse events associated with the first, it is better to use intra-articular LMW HA in patients with OA of the knee in clinical research or practice.

There is one study comparing low and high molecular weight HA following intra-articular administration in hip OA patients [27]. The authors concluded that both treatments produced a significant reduction in OA symptoms but there was no statistically significant difference between the two groups. This study had a small patient population (32 hips LMW HA and 24 hips HMW HA) and they did not use a power calculation to prove that they included enough patients.

Viscosupplementation, performed with the use of fluoroscopy or ultrasound guidance, is an effective treatment of hip OA and can be an alternative method to other conservative treatments or total hip arthroplasty [14,28,29].

Intra-articular injection of HA or its derivates into the hip joint is safe and well tolerated. However, local adverse reactions (hot, painful, swollen joint) were reported, typically occurring 24-72 hours, and mostly after injection of HMW HA (Hylan) in the knee [30-33]. Ten to thirty per cent of hip OA patients treated with intra-articular injections of HA reported adverse effects, which is slightly higher than in OA of the knee [13]. Some patients experienced transient hip pain after the infiltration but all of them fully recovered in the following days without any treatment or with the use of NSAIDs. Although a single case of septic arthritis was reported after multiple intra-articular injections [13], gout, pseudogout and chondrocalcinosis have not been reported after hip infiltrations with HA.

#### Corticosteroids

Due to their anti-inflammatory effects, CS have been used in the treatment of knee OA for many years. Intra-articular infiltrations of CS in the hip can also reduce pain, stiffness and disability. Clinical experience has shown that CS are very useful for the treatment of exacerbations of OA, but they do not influence the underlying process of OA. CS are useful for acute periods of pain in hip OA but this effect only lasts for some months [7,8,34]. A significant pain reduction at rest and during activity was seen at 3 and 12 weeks follow-up. The range of joint motion increased significantly for all directions and functionality improved significantly after the injection of CS [35,36].

It is well known that intra-articular injections of CS have some side effects, particularly after repeated injections. These include thinning of the cartilage, a higher risk of infection and weakening of the ligaments of the injected joint.

Two doses of CS (40 mg and 80 mg) were used in one study [34]. At 6 weeks, the benefits of the 40 mg and 80 mg doses in improving pain and stiffness of the hip were similar. However hip function did not improve with the 40 mg dose but only with the 80 mg dose [34].

In this study we will use Depomedrol 80 mg/2 ml.

#### Bupivacaine

Bupivacaine is a member of the amide group in the local anesthetic family and has a molecular weight of 342.9 g/mol. Because chondrocyte apoptosis has been implicated in the development of OA [37], it is important to determine whether bupivacaine has cytotoxic effects on articular chondrocytes. Chu et al (2006) showed that in vitro exposure of bovine articular chondrocytes to 0.5% bupivacaine was cytotoxic and that in cartilage with an intact surface bupivacaine caused less cell death [38]. The authors concluded that an intact articular surface may provide a partial barrier to cytoxicity due to bupivacaine.

A rabbit shoulder model showed no permanent impairment of cartilage function 3 months after intra-articular infusion of bupivacaine [39]. Cartilage metabolism was higher than before indicating a reparative ability to recover from the damage caused by bupivacaine. No difference in clinical outcome was seen between 0.5% bupivacaine and placebo [40,41]. After total knee arthroplasty, the use of bupivacaine reduced pain and the need for narcotics during the first 24 hours [41]. Microscopic evaluation of human articular chondrocytes showed more than 95% cell death after exposure to 0.5% bupivacaine for 30 minutes [38]. Chondrocytes exposed to 0.25% bupivacaine showed a time-dependent reduction in viability, with longer exposure resulting in more cellular death that continued even after removal of bupivacaine. Finally, exposure to 0.125% bupivacaine for up to 60 minutes did not influence the viability of chondrocytes in comparison to a saline solution. Thus, human chondrocytes may tolerate bupivacaine 0.125%. Large human joints show a peak absorption of bupivacaine within the first hour [42,43]. The combination of systemic absorption and either lavage fluid or effusion is sufficient to dilute the injected bupivacaine to a concentration of less than 0.125%, which reduces the potential for chondrotoxity [44]. In this study we will use Marcaine (0.125%/2 ml bupivacaine).

### Description of methodology

This is a randomized, masked-observer, controlled trial with three-armed parallel-group design. Patients meeting the inclusion/exclusion criteria will be randomized to one of the following treatment groups:

1. Infiltration of the hip joint with Hyaluronic Acid.

2. Infiltration of the hip joint with Corticosteroids.

3. Infiltration of the hip joint with 0.125% Bupivacaine.

A block randomization scheme, with a block size equal to 8, will be used for the randomization to avoid at random imbalances in group size. No stratification variables are considered in the randomization.

Efficacy parameters will be assessed at baseline and at the planned follow-up visits at 6 weeks, 3 months and 6 months after the injection.

### Planned Statistical Analysis & Sample size calculation

A linear regression model for repeated measures will be used to compare the evolution of the scores between the groups. More specifically, a direct likelihood approach will be adopted using an unstructured covariance matrix for the repeated measures (Molenberghs and Kenward, 2007, Section 14.4). The approach has the advantage that less stringent assumptions are made, not only with respect to the covariance structure, but also to the mechanism underlying the missing observations. The approach does not assume that all variances or all covariances will be equal (as is done in classical repeated-measures ANOVA or in a mixed model using only a random subject effect). Furthermore, the approach allows that the missingness depends on the observed values (the so-called missing at random assumption (MAR)), whereas an analysis restricted to patients with complete information assumes the missingness to be completely at random (MCAR) (G. Molenberghs and M.G. Kenward)[45].

The primary analysis is based on intention-to-treat, i.e. all patients who received an infiltration at baseline are included. Appropriate transformations will be considered if needed to meet the statistical assumptions of the regression model. Time point-specific comparisons between the groups of the changes with respect to baseline will be made based on this model. Confidence intervals (CI) of the change as well as the difference (at each timepoint) between groups will be constructed. To protect against an inflated type-I error (due to the presence of two outcomes and two pairwise comparisons), an alpha-level of 0.01 will be used for each comparison with respect to the control group (Bonferroni correction).

A sample size calculation was performed to have at least 80% power to detect in both groups compared with placebo, a clinically meaningful difference in change (baseline-6 months) of 10 mm in VAS and 10 points in HSS. The SD of HSS and VAS is assumed to be equal to 16 and 20 respectively. Estimates are obtained from Frihagen et al. [46] and Qvistgaard [15]. Assuming a moderate correlation of 0.5 between the measurement at baseline and after 6 months, a total of 285 patients are needed, i.e., 95 subjects in each group. With this sample size, 80%power is achieved for VAS and 96.2% for HHS. The number of included subjects will be increased by 10% to compensate for the loss of power due to patients with missing observations (therefore, 105 patients per group will be recruited). Interim analyses are planned after 1/3 and 2/3 of the patients included reach the end of the follow-up period to allow for an early stopping of the study (or accrual of patients in a specific treatment group) due to rejection of the null hypothesis. Using the O'Brien-Fleming method (O'Brien and Fleming 1979) results in respectively |4.495|, |3.178| and |2.595| as critical values for the Z-statistic at the three analysis time points. Otherwise stated, p-values are declared significant if they reach p < 0.000007, p < 0.00148 and p < 0.0095 at the first interim analysis, the second interim analysis and at the final analysis, respectively. (Note that the 'price to pay' for performing the interim analysis is a slightly more conservative alpha-level at the final analysis, i.e. alpha = 0.0095 instead of alpha = 0.01)

#### Primary outcomes

Assessment of group differences at 6 months for:

• Harris Hip Score (HHS) [18]: this score will be used as a self-administered questionnaire in accordance with the developers' instructions. The Harris Hip Score was first developed in 1967 and is accepted as one of the best used questionnaires dealing with hip function. It is a disease-specific scoring system which was introduced to provide an evaluation system for various hip disabilities and methods of treatment. This Score gives a maximum of 100 points, with domains of pain, function, deformity and motion. Pain and function are the two basic considerations and receive the heaviest weighting (44 and 47 points, respectively). Range of motion and deformity are seldom of primary importance and therefore receive five and four points, respectively. Function is subdivided into activity of daily living (ADL, 14 points) and gait (33 points).

• Visual Analog Score (VAS) [19]: this score is a self-assessment of variation in pain intensity, measured on a simple 100-mm-long continuous scale of absolutes ranging from 0 = "no pain" to 100 = "extreme pain". The percentage of pain is determined by physically measuring from the "0" end to the patients' mark on the pain scale and dividing this value by total length of the line. The advantage of the VAS is that you can determine the change in pain by taking the difference between any two recordings of pain severity.

#### Secondary outcomes

Assessment of group differences on:

• Hip disability and Osteoarthritis Outcome Score (HOOS) [20]: this score will be used as a self-administered questionnaire with the help of the developers' instructions. The HOOS is an adaptation of the Knee disability and Osteoarthritis Outcome Score (KOOS) intended to evaluate symptoms and functional limitations related to the hip. The HOOS consists of 40 items assessing five different patient-relevant groups: Pain (P) (ten items); Symptoms (S) including stiffness and range of motion (five items); Activity limitations-daily living (A) (seventeen items); Sport and Recreation Function (SP) (four items); and Hip Related Quality of Life (Q) (four items). The HOOS contains all Western Ontario and McMaster Universities Osteoarthritis Index (WOMAC) questions in unchanged form. The WOMAC scores are often used in scientific literature and it is important that if needed the WOMAC scores can be calculated from the HOOS questionnaire. To answer each question, five options are used (no, mild, moderate, severe, extreme). All items are scored from zero to four, and each of the five subscales will be calculated as the sum of the items included. The HOOS is transformed into a 0-100 worst to best score.

• VAS, Harris Hip Score: at all follow-up time points, except after 6 months

• Patient evaluation of treatment outcome: The patients will be asked to assess their status on a 3-point scale (worse, stable or better then at the time of enrollment).

• Intake of escape medication: The number of escape medication taken/day will be determined.

• Safety: All complications/adverse events will be evaluated and the relationship to the test product determined. When they have complications, patients will be seen in our department earlier than normal.

The above primary and secondary measure instruments will be assessed at the time of enrollment into the study prior to any injection (baseline), and then again at six weeks, 3 and 6 months after the injection. The six-month follow-up period for all patients will begin on the date the injection is administered.

### Ethical review committee and informed consent requirements

Ethical approval was necessary because this is a prospective randomized study. Ethical approval has been obtained from the ethical committee of the Catholic University, Leuven. Informed consent forms will be signed by the patients before inclusion into our study. If patients, after reading the patient information, have any questions about our study protocol, they can contact Dr. Sascha Colen or Dr. Michiel Mulier.

## Discussion

This study is primarily designed to evaluate the effectiveness of non-operative treatment of hip OA using HA and CS infiltrations. A comparison is made between HA, CS and bupivacaine (control group) infiltrations. There have been studies and even randomized clinical trials on non-operative treatment of hip OA but the methodological quality is often poor. There is a need for more rigorously designed studies on hip OA treatment as this subject is still very much under debate. By publishing our protocol we wish to share the robust design and methodological quality of our protocol. Moreover, when the design of a study is published it will help to achieve transparency about why and how studies are undertaken. The publication of a study design may help to reduce the problem of publication bias, i.e. selective publication of positive associations and disregarding negative and weak associations, prevent unnecessary duplication of research efforts and duplicate publication. To our knowledge, there has never been a similar study design published on the same subject. By making this study design available we wish to contribute to a more in-depth research on non-operative treatment of osteoarthritis of the hip and prevent publication bias for this double blinded randomized trial. Results of the trial will be disseminated through publication in relevant peer-reviewed journals and conference proceedings.

## Authors' contributions

All authors were involved in the design of the study. For all of them there are no competing interests according to the products used. SC was responsible for drafting the paper, and all authors commented on the draft. All authors have read and approved the final manuscript.

## Pre-publication history

The pre-publication history for this paper can be accessed here:

http://www.biomedcentral.com/1471-2474/11/264/prepub
